# Luminescence of SiO_2_-BaF_2_:Tb^3+^, Eu^3+^ Nano-Glass-Ceramics Made from Sol–Gel Method at Low Temperature

**DOI:** 10.3390/nano12020259

**Published:** 2022-01-14

**Authors:** Natalia Pawlik, Barbara Szpikowska-Sroka, Tomasz Goryczka, Ewa Pietrasik, Wojciech A. Pisarski

**Affiliations:** 1 Institute of Chemistry, University of Silesia, 40-007 Katowice, Poland; barbara.szpikowska-sroka@us.edu.pl (B.S.-S.); ewa.pietrasik@us.edu.pl (E.P.); 2 Institute of Materials Engineering, University of Silesia, 41-500 Chorzow, Poland; tomasz.goryczka@us.edu.pl

**Keywords:** BaF_2_ nanophase, oxyfluoride nano-glass-ceramics, Tb^3+^/Eu^3+^ energy transfer, sol–gel chemistry

## Abstract

The synthesis and characterization of multicolor light-emitting nanomaterials based on rare earths (RE^3+^) are of great importance due to their possible use in optoelectronic devices, such as LEDs or displays. In the present work, oxyfluoride glass-ceramics containing BaF_2_ nanocrystals co-doped with Tb^3+^, Eu^3+^ ions were fabricated from amorphous xerogels at 350 °C. The analysis of the thermal behavior of fabricated xerogels was performed using TG/DSC measurements (thermogravimetry (TG), differential scanning calorimetry (DSC)). The crystallization of BaF_2_ phase at the nanoscale was confirmed by X-ray diffraction (XRD) measurements and transmission electron microscopy (TEM), and the changes in silicate sol–gel host were determined by attenuated total reflectance infrared (ATR-IR) spectroscopy. The luminescent characterization of prepared sol–gel materials was carried out by excitation and emission spectra along with decay analysis from the ^5^D_4_ level of Tb^3+^. As a result, the visible light according to the electronic transitions of Tb^3+^ (^5^D_4_ → ^7^F_J_ (J = 6–3)) and Eu^3+^ (^5^D_0_ → ^7^F_J_ (J = 0–4)) was recorded. It was also observed that co-doping with Eu^3+^ caused the shortening in decay times of the ^5^D_4_ state from 1.11 ms to 0.88 ms (for xerogels) and from 6.56 ms to 4.06 ms (for glass-ceramics). Thus, based on lifetime values, the Tb^3+^/Eu^3+^ energy transfer (ET) efficiencies were estimated to be almost 21% for xerogels and 38% for nano-glass-ceramics. Therefore, such materials could be successfully predisposed for laser technologies, spectral converters, and three-dimensional displays.

## 1. Introduction

Barium fluoride, BaF_2_, belongs to the group of attractive nanoparticles, produced using different preparation methods and applied in numerous multifunctional applications. Nd^3+^:BaF_2_ nanocrystals synthesized by the reverse microemulsion technique present interesting luminescence properties [1]. Indeed, the quenching of fluorescence intensity (λ_em_ = 1052 nm) in nanosized Nd^3+^:BaF_2_ domains was not observed even under very high dopant levels (~45 mol.% of Nd^3+^). Further experiments revealed the crystallization of cubic and orthorhombic BaF_2_ nanoparticles, and it was proven that such fluoride crystals could be quite easily transformed from the orthorhombic phase to the more thermodynamically stable cubic phase under certain preparation conditions. This effect was confirmed by X-ray diffraction (XRD) and high-resolution transmission electron microscopy (HR-TEM) used for self-assembled monodisperse BaF_2_ nanocrystals accomplished by the liquid–solid-solution (LSS) approach [2]. BaF_2_ nanocrystals were also fabricated from precursor Na_2_O-K_2_O-BaF_2_-Al_2_O_3_-SiO_2_ glasses via their controlled heat treatment. Their self-organized nanocrystallization processes [3] and size distribution [4] have been presented and discussed in detail. Luminescence properties of nanosized Eu^3+^-doped BaF_2_ synthesized via an ionic liquid-assisted solvothermal method in different solvents (e.g., DMSO, water, or water with PVP solution) confirmed that these fluoride nanoparticles can be effectively used for bioimaging applications [5].

From the accumulated experience and literature data, it is known that RE^3+^ ions can be introduced into the fluoride nanocrystals dispersed within the transparent glassy host. Indeed, several precursor glasses doped with RE^3+^ ions were heat treated to fabricate RE^3+^:BaF_2_ nanocrystals and obtain transparent glass-ceramics with enhanced luminescence properties. Nano-glass-ceramics with RE^3+^:BaF_2_ have been examined for visible [6] and near-infrared [7,8] luminescence as well as white up-conversion applications [9]. Special attention has been devoted to the structure and luminescent properties of BaF_2_ nanocrystals in glass-ceramics singly doped with Er^3+^ [10,11] and co-doped with Er^3+^/Yb^3+^ [12,13]. Among RE^3+^, trivalent europium ions are commonly used as a spectroscopic probe, indicating structural changes around the optically active ions and their surrounding environment [14]. Additionally, europium ions in a divalent oxidation state can also exist, thus, the silicate glasses containing EuF_3_ synthesized by melt-quenching in reducing atmosphere tend to form Eu^2+^-doped glass-ceramics after the heat-treatment process. The prepared glass-ceramic system with Eu^2+^:BaF_2_ nanocrystals could be potentially utilized as a blue phosphor for UV–LED applications [15]. Divalent europium ions in fluorosilicate glass-ceramics can be well stabilized via lattice site substitution [16]. In the field of preparation of the RE^3+^-doped glass-ceramics containing BaF_2_ nanocrystals, particular attention should also be focused on the sol–gel method. The first synthesis of 95SiO_2_−5BaF_2_ (mol.%) nano-glass-ceramics via the sol–gel technique was reported and described in work by D. Chen et al. [17]. The authors proved that the size of precipitated BaF_2_ nanocrystals (2–15 nm) and the luminescence of Er^3+^ ions are strictly dependent on heat-treatment conditions of initially obtained xerogels. C.E. Secu et al. [18,19] presented the fabrication, structure, and luminescence of 95SiO_2_–5BaF_2_ (mol.%) nano-glass-ceramics singly doped with Pr^3+^, Ho^3+^, Dy^3+^, Sm^3+^, and Eu^3+^ ions. Except for Eu^3+^-doped samples, the emission bands of other active dopants were revealed after controlled heat treatment of precursor xerogels, which was explained by incorporating RE^3+^ into BaF_2_ crystals (3–7 nm) and removing residual OH groups from the silicate sol–gel host. The recently published work by M. Hu et al. [20] was concentrated on properties of 95SiO_2_–5BaF_2_ (mol.%) glass-ceramics singly and doubly doped by Tb^3+^, Eu^3+^, and Dy^3+^ ions, containing fluoride nanocrystals with an average size of ~5 nm. The authors verified the thermal stability of generated luminescence in a range from 30 °C to 290 °C, proving that synthesized sol–gel nano-glass-ceramics could be utilized as color and white light emitters. The properties of RE^3+^-doped sol–gel glass-ceramics containing BaF_2_ nanocrystals were compared with other oxyfluoride systems in an extensive review published recently by Secu et al. [21]. This class of RE^3+^-doped materials is widely considered as a promising candidate for selected applications, e.g., three-dimensional displays, flat color screens, spectral converters, light-emitting diodes (LEDs), etc. [21]. 

Our previously published work [22] was concerned with sol–gel SiO_2_-BaF_2_ nano-glass-ceramic systems doped with europium ions in a trivalent oxidation state. Their structural and optical properties have been studied using various experimental techniques, such as differential scanning calorimetry (DSC), X-ray diffraction (XRD), transmission electron microscopy (TEM) coupled with the energy-dispersive X-ray spectroscopy (EDS), infrared (ATR-IR), and luminescence spectroscopy. The properties of Tb^3+^, Eu^3+^ co-doped glass-ceramic systems containing BaF_2_ nanocrystals made from the sol–gel method at a low temperature are communicated here. To the best of our knowledge, these aspects for SiO_2_-BaF_2_:Tb^3+^, Eu^3+^ nano-glass-ceramics have not yet been examined.

## 2. Materials and Methods

The xerogels singly doped with Tb^3+^ and co-activated with Tb^3+^, Eu^3+^ ions were prepared via the previously described sol–gel synthesis [22]. The reagents from Sigma-Aldrich (St. Louis, MO, USA) were applied for the fabrication the samples. In the first step of preparation, precursor (TEOS), ethanol, deionized water, and acetic acid (AcOH) were mixed (in a molar ratio 1:4:10:0.5) in round-bottom flasks for 30 min. TEOS, Si(OC_2_H_5_)_4_, was used as a precursor for creating the SiO_2_ silicate host, water was necessary to perform the hydrolysis reaction of TEOS, and AcOH played a role as a catalyst. Due to the significantly limited solubility of TEOS in water, ethyl alcohol was introduced into the reaction systems, enabling the hydrolysis reaction by increasing the TEOS–water contact surface. The hydrolysis could be expressed by the following reaction:

Si(OC_2_H_5_)_4_ + nH_2_O ⇄ Si(OH)_n_(OC_2_H_5_)_(4-n)_ + nC_2_H_5_OH,

in which n ≤ 4. Simultaneously with the hydrolysis reaction, the condensation begins, which allows for the creation of a silicate network through the formation of siloxane bridges, Si–O–Si. The homocondensation could be given by the following chemical reaction:

(OC_2_H_5_)_n_(OH)_(3-n)_Si–OH + HO–Si(OC_2_H_5_)_n_(OH)_(3-n)_ ⇄ (OC_2_H_5_)_n_(OH)_(3-n)_Si–O–Si(OC_2_H_5_)_n_(OH)_(3–n)_ + H_2_O,

and the heterocondensation could be expressed by:

(OC_2_H_5_)_n_(OH)_(3–n)_Si–OH + C_2_H_5_O–Si(OC_2_H_5_)_n_(OH)_(3–n)_ ⇄ (OC_2_H_5_)_n_(OH)_(3–n)_Si–O–Si(OC_2_H_5_)_n_(OH)_(3–n)_ + C_2_H_5_OH,

in which n ≤ 3. The mechanisms of hydrolysis and condensation reactions of alkoxides were discussed in detail in the paper [23].

After pre-hydrolysis and pre-condensation, the solutions of Ba(AcO)_2_ and RE(AcO)_3_ (RE = Tb or Tb/Eu) in trifluoroacetic acid (CF_3_COOH, TFA) and deionized water were added dropwise, and the obtained mixtures were stirred for the next 60 min. Since the electrolytic dissociation of TFA acid (K_a_ = 5.9 × 10^−1^) is greater than for AcOH (K_a_ = 1.8 × 10^−5^), TFA is a much stronger acid than AcOH, and the following reaction occurs:

Ba(AcO)_2_ + 2TFA → Ba(TFA)_2_ + 2AcOH.

For Tb^3+^-doped samples, the molar ratio of TFA:Ba(AcO)_2_:Tb(AcO)_3_ was equal to 5:0.95:0.05, and for Tb^3+^, Eu^3+^ co-doped materials, the molar ratio of TFA:Ba(AcO)_2_:Tb(AcO)_3_:Eu(AcO)_3_ was equal to 5:0.9:0.05:0.05. The mass of TEOS, ethanol, deionized water, and acetic acid reached 90 wt.% of each sample, and the mass of the remaining part containing TFA, Ba(AcO)_2_, and RE(AcO)_3_ (RE = Tb or Tb/Eu) equaled 10 wt.%. The liquid sols were dried at 35 °C for several weeks and then heat treated at 350 °C per 10 h in a muffle furnace (Czylok, Jastrzębie-Zdrój, Poland). The thermal treatment of xerogels at 350 °C aims to transform them into SiO_2_-BaF_2_ nano-glass-ceramics. Indeed, TFA was introduced as a fluorination reagent, allowing for successful crystallization of BaF_2_ fraction inside the silicate sol–gel host. The fabricated xerogels were denoted as XG_Tb_ and XG_Tb/Eu_ (for singly and doubly doped xerogels), as well as nGC_Tb_ and nGC_Tb/Eu_ (for singly and co-doped nano-glass-ceramics).

The thermogravimetry and differential scanning calorimetry (TG/DSC) were carried out using a Labsys Evo system with a heating rate of 10 °C/min in argon atmosphere (SETARAM Instrumentation, Caluire, France). To verify the formation of fluoride nanocrystals within the silicate sol–gel host at 350 °C, the X-ray diffraction was performed using an X’Pert Pro diffractometer equipped by PANalytical with CuK_α_ radiation (Almelo, the Netherlands). Additionally, the fluoride nanocrystals were observed by a JEOL JEM 3010 transmission electron microscope operated at 300 kV (JEOL, Tokyo, Japan). The structural characterization was supplemented by infrared spectroscopy (IR). The experiment was performed with the use of the Nicolet iS50 ATR spectrometer (Thermo Fisher Scientific Instruments, Waltham, MA, USA), and the spectra were collected in attenuated total reflectance (ATR) configuration within the 4000–400 cm^−1^ as well as 500–200 cm^−1^ ranges (64 scans, 4 cm^−1^ resolution).

The luminescence measurements were performed on a Photon Technology International (PTI) Quanta-Master 40 (QM40) UV/VIS Steady State Spectrofluorometer (Photon Technology International, Birmingham, NJ, USA) supplied with a tunable pulsed optical parametric oscillator (OPO) pumped by the third harmonic of a Nd:YAG laser (Opotek Opolette 355 LD, OPOTEK, Carlsbad, CA, USA). The laser system was coupled with a 75 W xenon lamp, a double 200 mm monochromator, and a multimode UV/VIS PMT (R928) (PTI Model 914) detector. The excitation and emission spectra were recorded with a resolution of 0.5 nm. The luminescence decay curves were recorded by a PTI ASOC-10 (USB-2500) oscilloscope. All structural and optical measurements were carried out at room temperature.

## 3. Results and Discussion

### 3.1. Thermal Behavior of Synthesized Xerogels

**Figure 1:**
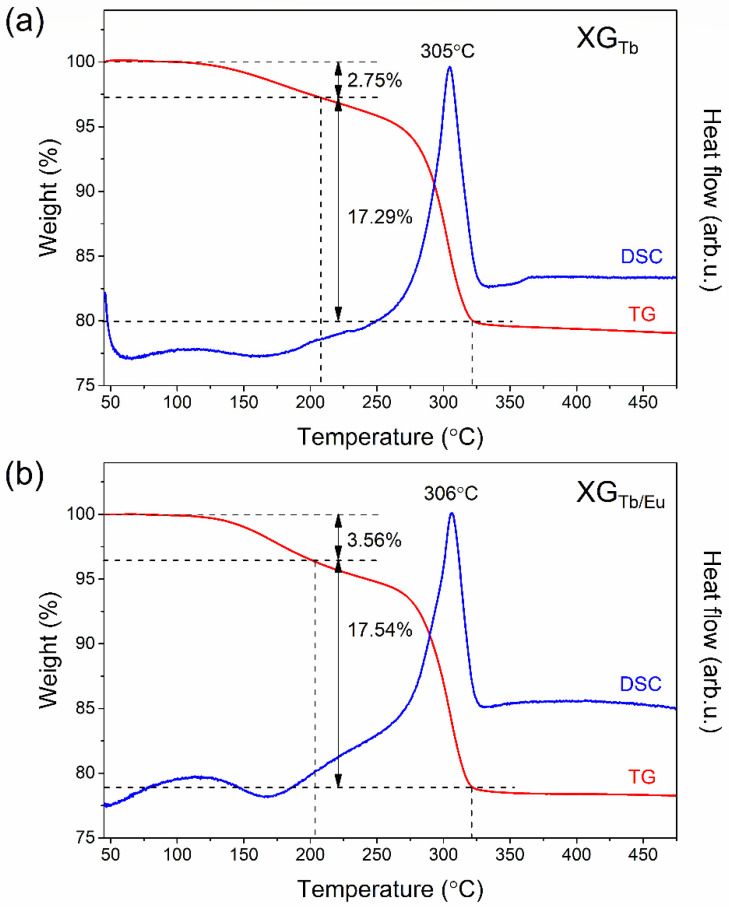
DSC and TG curves of prepared xerogels singly doped with Tb^3+^ (**a**), and co-doped with Tb^3+^/Eu^3+^ ions (**b**).

Figure 1 presents the TG/DSC curves recorded for fabricated xerogels in an inert gas atmosphere in a temperature range from 45 °C to 475 °C (the heating rate during measurement was 10 °C/min). According to TG curves, there are two distinguishable degradation steps for both fabricated samples: first, identified at 45–(~205) °C, and second, observed in the temperature range (~205) °C–(~320) °C. A slight weight loss, about 2.75% (XG_Tb_) and 3.56% (XG_Tb/Eu_), is associated with the elimination of residual solvents (ethyl alcohol, acetic acid) and water from the porous sol–gel host. At higher temperatures, strong exothermic peaks with maxima at 305 °C (XG_Tb_) and 306 °C (XG_Tb/Eu_) were identified, which appear along with ~17.55% weight loss. Generally, trifluoroacetates tend to decompose at temperatures near ~300 °C, which is well documented and described in the current literature [24,25,26]. Thus, recorded exothermic DSC peaks are clearly correlated with thermal decomposition of Ba(TFA)_2_ and crystallization of BaF_2_, which could be given by the chemical reaction:

$$\mathrm{Ba}(\mathrm{TFA}{)}_{2}\overset{T}{\to }{\mathrm{BaF}}_{2}+{\mathrm{CF}}_{3}\mathrm{CFO}+{\mathrm{CO}}_{2}+\mathrm{CO}.$$

The thermolysis led to cleavage of C–F bonds from −CF_3_ groups, and the resultant fluorine ions (F^−^) tend to react with Ba–O bonds, forming BaF_2_ phase [27]. The heat exchanged during degradation of Ba(TFA)_2_ in studied sol–gel materials is close to −118 J/g. J. Farjas et al. [28] pointed out that the denoted heat exchange during the degradation of trifluoroacetates depends on atmosphere (air or ambient gas) and the presence of vapored water. Our obtained value is comparable with DSC results obtained for pure Ba(TFA)_2_ salt in argon atmosphere [28]. The data obtained from TG/DSC analysis for the studied sol–gel samples are shown in Table 1 and Table 2.

### 3.2. Structural Characterization by XRD, TEM, and ATR-IR

**Figure 2:**
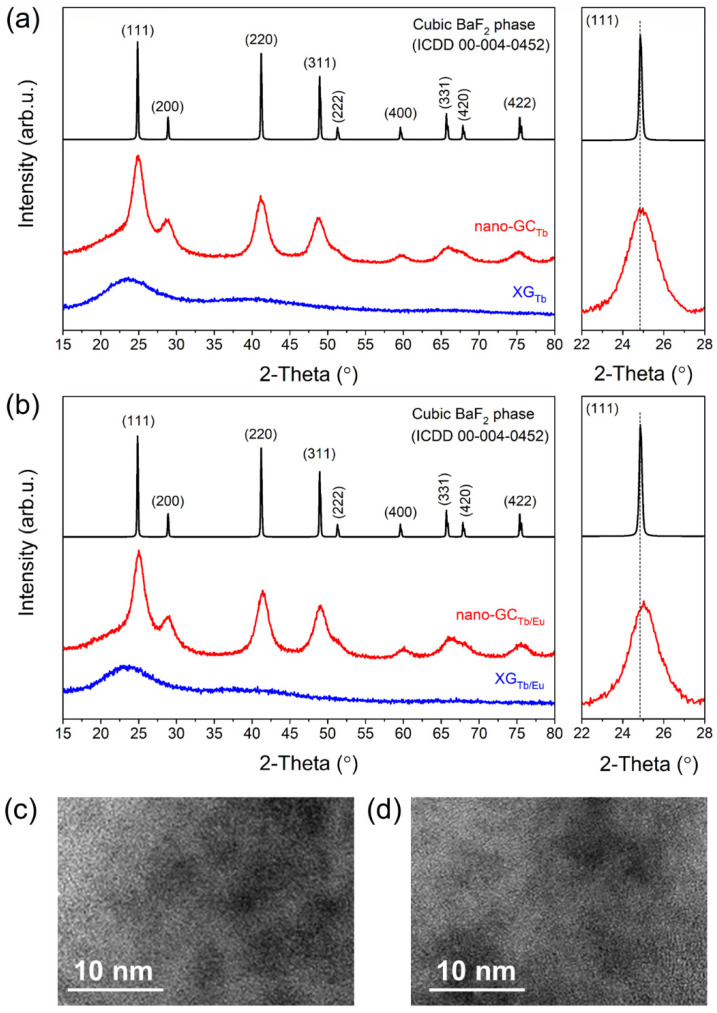
XRD patterns of prepared sol–gel samples: Tb^3+^ singly doped materials (**a**) and Tb^3+^, Eu^3+^ co-doped specimens (**b**). The standard data for the BaF_2_ cubic phase (ICDD card no. 00-004-0452) are also shown for comparison. TEM images revealed the presence of fluoride crystals in glass-ceramics singly doped with Tb^3+^ (**c**) and co-doped with Tb^3+^, Eu^3+^ (**d**).

Figure 2 presents the X-ray diffraction (XRD) patterns of the xerogels and nano-glass-ceramics fabricated at 350 °C. The diffractograms collected for Tb^3+^ singly doped samples are depicted in Figure 2a, meanwhile, the data for Tb^3+^, Eu^3+^ co-doped materials are shown in Figure 2b. The XRD patterns of the precursor xerogels revealed any sharp diffraction lines, but only a broad hump with a maximum at ~25°, indicating their amorphous nature without long-range order [29]. Conversely, the intense diffraction lines were observed only after thermal treatment of xerogels at 350 °C for 10 h. The XRD patterns of prepared glass-ceramics are in accordance with the standard diffraction lines of cubic BaF_2_ crystallized in the Fm3m space group (ICDD card no. 00-004-0452), confirming the precipitation of fluoride crystals inside the silicate sol–gel matrix. The crystalline size of BaF_2_ in fabricated glass-ceramics was evaluated by calculations with the Scherrer formula given below [30]:

(1) $$D=\frac{K\lambda }{B\cos\theta }$$

in which K is a shape factor (in our calculations, K = 1 was taken), λ is a wavelength of X-rays (0.154056 nm, Kα line of Cu), Β is a broadening of the diffraction peak at half the maximum intensity, and θ is Bragg’s angle. The average crystal sizes of BaF_2_ were calculated to be 5 nm ± 0.1 nm for both nGC_Tb_ and nGC_Tb/Eu_ samples. The average size of BaF_2_ nanocrystallites was also calculated from a Williamson–Hall plot as follows [31]:

(2) $$D=\frac{K\lambda }{\beta \cos\theta }$$

where β is half of the width of the diffraction line, whereas (Δa/a) refers to the lattice deformation. 

The Scherrer method makes the half-width of the diffraction line dependent only on the size of the crystallites. On the other hand, in the Williamson–Hall analysis, the internal stresses reflected by the lattice deformation are additionally taken into account in the broadening of the diffraction line. The mean crystal sizes, calculated with the Williamson–Hall method, were estimated to be 4.3 nm ± 0.1 nm for nGC_Tb_, and 4.8 nm ± 0.1 nm for nGC_Tb/Eu_. Moreover, the lattice deformation was negligible and less than 0.1%. The obtained results of the average crystallite size, from both methods, reveal good agreement. They proves the lack of internal stresses in the formed BaF_2_ particles. The crystal lattice parameters for BaF_2_ phase were determined to be 6.188 (8) Å (Tb^3+^-doped sample) and 6.169 (7) Å (Tb^3+^, Eu^3+^ co-doped sample), which are slightly smaller than the lattice parameter for undoped barium fluoride (a_0_ = 6.2001 Å). Indeed, both Tb^3+^ (1.04 Å) and Eu^3+^ (1.07 Å) ions [32] with smaller ionic radii could substitute Ba^2+^ (1.35 Å) [33] cations in BaF_2_ crystal lattice, resulting in a decrease in the unit cell volume. The indicated changes in the lattice parameter are also noticeable as a slight shift of the recorded diffraction lines towards higher values of the 2θ angle (an enlargement within a 22–28° angle, in which the (111) diffraction line was detected; shown in Figure 1). Additionally, it was observed that the shift of diffraction lines is more clearly visible for the nGC_Tb/Eu_ sample, which evidences that the total incorporation of RE^3+^ ions inside BaF_2_ nanocrystals is higher than for nGC_Tb_. Similar results from XRD measurements were described in the literature for other oxyfluoride optical systems, e.g., SiO_2_-LaF_3_:Er^3+^ sol–gel nano-glass-ceramics [34] and germano-gallate glass-ceramics containing BaF_2_:Er^3+^ nanocrystals [11]. Figure 2c,d display the TEM images of prepared nano-glass-ceramic samples singly doped with Tb^3+^ and co-doped with Tb^3+^, Eu^3+^ ions, respectively. The size of BaF_2_ nanocrystals was average, estimated from the Scherrer equation and the Williamson–Hall method.

**Figure 3:**
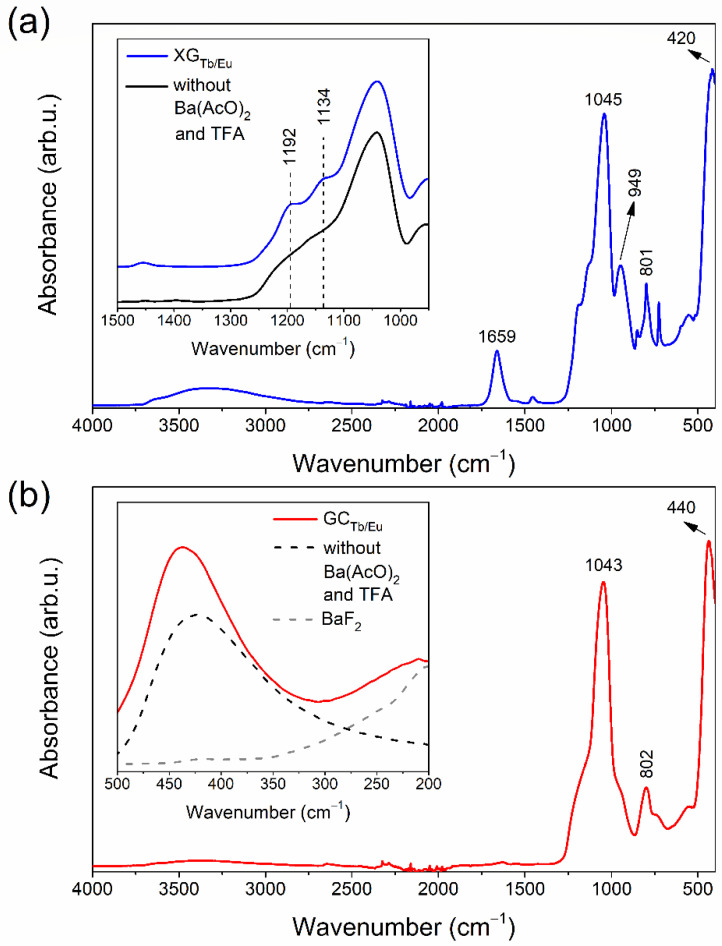
ATR-IR spectra recorded for xerogel XG_Tb/Eu_ (**a**) and nano-glass-ceramic nGC_Tb/Eu_ (**b**) co-doped with Tb^3+^, Eu^3+^ ions.

The ATR-IR spectrum within the 4000–400 cm^−1^ range for a representative XG_Tb/Eu_ sample is shown in Figure 3a, and the assignment of individual IR peaks was carried out based on the literature [35,36]. The recorded infrared signals confirmed the formation of a polycondensed silicate network created by Q^2^ (949 cm^−1^), Q^3^ (1045 cm^−1^), and Q^4^ (1134 cm^−1^) units of SiO_2_ tetrahedrons as well as Si–O–Si siloxane bridges (1192 cm^−1^, 801 cm^−1^). On the other hand, the signals recorded at ~3665 cm^−1^ and ~3400 cm^−1^, according to vicinal/geminal and hydrogen-bonded Si–OH groups, respectively, clearly pointed to the presence of unreacted silanol groups. Indeed, xerogels are highly porous materials [37], hence, the IR bands originating from Si–OH groups are expected. Indeed, the next recorded infrared band, 1659 cm^−1^, revealed the vibrations of Si–OH groups, but also oscillations within C=O carbonyl groups (from residual AcOH and unreacted TFA), as well as molecular water. A signal at ~3200 cm^−1^ was interpreted as vibrations from hydrogen-bonded OH groups in organic compounds and water, and may confirm that pores inside the silicate network are filled with liquids. It should be noted that peaks located near ~1134 cm^−1^ and ~1192 cm^−1^ could be assigned, despite Q^4^ units and Si–O–Si bridges, to oscillations of C–F bonds in Ba(TFA)_2_ and unreacted TFA. Indeed, a comparison of ATR-IR spectra in this region for XG_Tb/Eu_ xerogel and an analogous sample prepared without the addition of Ba(AcO)_2_ and TFA revealed that the signals are more intense for XG_Tb/Eu_ (inset of Figure 3a). This could point to the presence of additional oscillators that contribute to overall signals recorded at ~1134 cm^−1^ and ~1192 cm^−1^. The signal recorded at ~420 cm^−1^ was assigned to the O–Si–O bending vibration.

The ATR-IR spectrum within the 4000–400 cm^−1^ region registered for a representative nGC_Tb/Eu_ sample obtained at 350 °C is shown in Figure 3b. Compared with the ATR-IR spectrum for XG_Tb/Eu_, the intensities of signals at >3000 cm^−1^ and 1659 cm^−1^ weakened significantly, which allows us to make conclusions about evaporation of volatile chemical components from the sol–gel host and progressive reactions between unreacted Si–OH groups. Additionally, it was also observed that for the nGC_Tb/Eu_ nano-glass-ceramic sample, the intensity of the IR signal near ~949 cm^−1^ is weaker than for the XG_Tb/Eu_ xerogel sample. An indicated effect may also suggest a continuation of polycondensation because Q^2^ units probably transformed into Q^3^ and Q^4^ ones, which favor the creation of a more cross-linked sol–gel host. It was also observed that intensities of IR signals near ~1134 cm^−1^ and ~1192 cm^−1^ weakened compared with those recorded for the xerogel. This effect could be explained by thermal decomposition of Ba(TFA)_2_ compound into BaF_2_ crystals within the prepared silicate sol–gel host in the proposed heat-treatment conditions. Indeed, the Ba–F vibrations might be observed at lower frequencies (inset of Figure 3b), which agrees with the IR spectrum recorded for pure BaF_2_ [38]. The peak with a maximum at ~440 cm^−1^ was recorded for both nGC_Tb/Eu_ nano-glass-ceramics and an analogous sample prepared without the addition of Ba(AcO)_2_ and TFA, confirming that such a band is not related to the fluoride fraction but to the oscillations within the silicate host (O–Si–O vibration). It should be noticed that this band shifts toward a higher frequency for nano-glass-ceramics in comparison with the xerogel. The reason for such spectral behavior could be explained by differences in the inter-tetrahedra angle of SiO_4_ units in xerogels and glass-ceramics, as was stated in the literature [39].

### 3.3. Luminescence of Amorphous Silicate Xerogels

**Figure 4:**
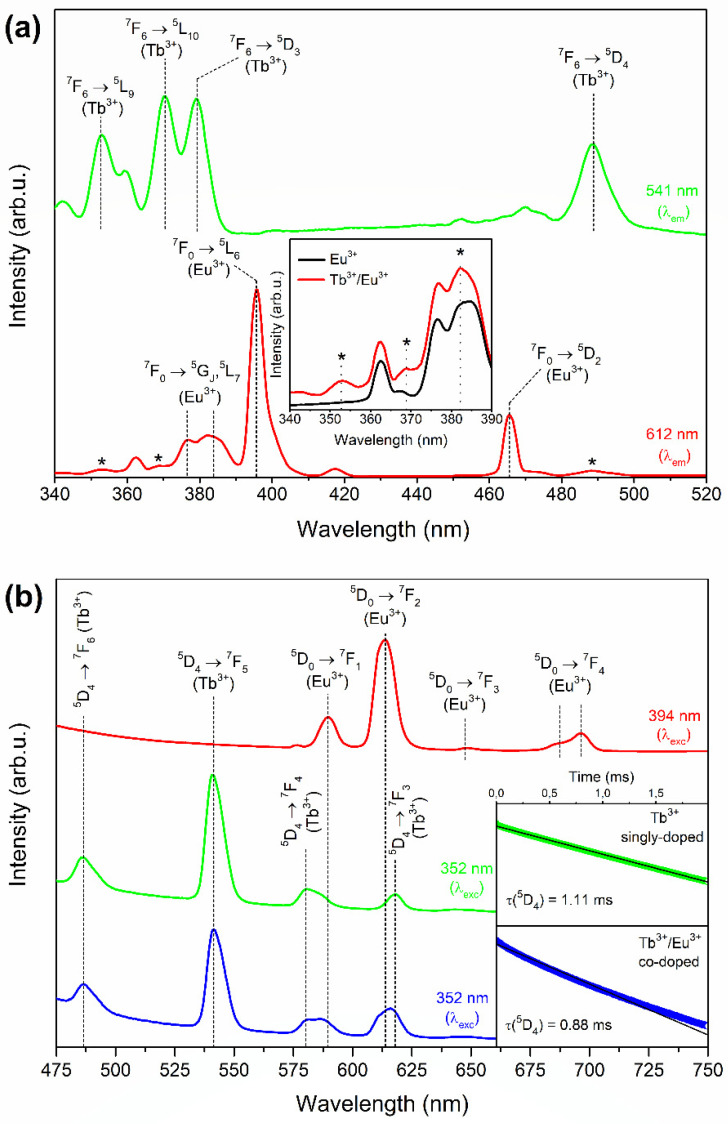
The excitation spectra recorded for Tb^3+^ (λ_em_ = 541 nm) and Eu^3+^ (λ_em_ = 612 nm) ions in fabricated amorphous xerogels. For the latter, the additional lines originated from Tb^3+^ ions were marked by asterisks (**a**). The registered luminescence spectra collected for XG_Tb_ (green line, λ_exc_ = 352 nm) and XG_Tb/Eu_ samples (blue line, λ_exc_ = 352 nm; red line, λ_exc_ = 394 nm). Inset shows the decay curves recorded for the ^5^D_4_ state of Tb^3+^ ions (**b**).

Figure 4a shows the excitation spectra of the prepared XG_Tb/Eu_ samples. The spectra were registered within the 340–520 nm spectral range on collecting the luminescence at 541 nm and 612 nm wavelengths. The excitation spectrum, while monitoring the green emission line at 541 nm, revealed the characteristic bands for Tb^3+^ ions according to the following transitions within the near-UV and VIS scope: ^7^F_6_ → ^5^L_9_ (352 nm), ^7^F_6_ → ^5^L_10_ (370 nm), ^7^F_6_ → ^5^D_3_ (379 nm), and ^7^F_6_ → ^5^D_4_ (488 nm). Meanwhile, the spectrum recorded by collecting the red luminescence at 612 nm showed the excitation lines of Eu^3+^ related to the electronic transitions from the ^7^F_0_ ground level into the following excited states: ^5^G_J_ (376 nm), ^5^L_7_ (384 nm), ^5^L_6_ (394 nm), ^5^D_3_ (418 nm), and ^5^D_2_ (464 nm). However, it was observed that the spectrum recorded for XG_Tb/Eu_ contains some additional weak bands, which did not appear for the sample singly doped with Eu^3+^ (for better visibility, an enlargement of the 340–390 nm scope is presented in the inset of Figure 2a, and the bands are marked by asterisks). It should be noted that the recorded additional bands correspond to the contribution of excitation lines originating from Tb^3+^ ions (^7^F_6_ → ^5^L_9_ (352 nm), ^7^F_6_ → ^5^L_10_ (379 nm), and ^7^F_6_ → ^5^D_4_ (488 nm)). Moreover, a slight shift of the ^7^F_0_ → ^5^L_7_ band (from 384 nm to 382 nm) was also denoted, which could be related to its overlapping with the ^7^F_6_ → ^5^D_3_ excitation line originating from Tb^3+^ co-dopant. Hence, the obtained results could suggest the occurrence of Tb^3+^ → Eu^3+^ ET. A similar interpretation of excitation spectra was described for lead borate glasses co-doped with Tb^3+^ and Eu^3+^ ions [40].

The fluorescence spectra of prepared sol–gel specimens are displayed in Figure 4b. The emission spectrum recorded for the XG_Tb/Eu_ sample under excitation at 394 nm (presented as a red line) consisted of several emission lines at 574 nm (^5^D_0_ → ^7^F_0_), 590 nm (^5^D_0_ → ^7^F_1_), 612 nm (^5^D_0_ → ^7^F_2_), 648 nm (^5^D_0_ → ^7^F_3_), and 696 nm (^5^D_0_ → ^7^F_4_) within the reddish-orange light area. It was observed that the ^5^D_0_ → ^7^F_2_ red emission band is the most prominent luminescence line, and the spectrum is similar to other Eu^3+^-doped typical glassy-like optical materials described in the literature [41,42]. Based on the collected spectrum, the R/O ratio (red-to-orange) was calculated using the areas of the ^5^D_0_ → ^7^F_2_ (R) and the ^5^D_0_ → ^7^F_1_ (O) bands. The R/O ratio value estimated for precursor silicate xerogel is relatively high and equals 3.92. It indicates that Eu^3+^ ions are far from an inversion center, which is characteristic for amorphous materials. In the luminescence spectrum of the XG_Tb_ sample (marked as a green line), the bands centered at 486 nm, 541 nm, 580 nm, and 618 nm were attributed to the ^5^D_4_ → ^7^F_J_ (J = 6–3) electronic transitions, respectively.

To verify the occurrence of ET between Tb^3+^ and Eu^3+^ ions in the studied silicate xerogels, the emission spectrum for the XG_Tb/Eu_ sample was recorded upon excitation at a 352 nm wavelength (shown as a blue line). The spectrum consisted of the following emission bands in the VIS spectral range: blue (486 nm), an intense green (541 nm), yellowish-orange (584 nm), and red (616 nm). The same bands within the blue–green light area were detected for the XG_Tb_ xerogel, and the mentioned emission lines were ascribed to the ^5^D_4_ → ^7^F_6_ and the ^5^D_4_ → ^7^F_5_ electronic transitions, respectively. Although the positions of these emission bands are the same, their intensity is slightly lower for the co-doped XG_Tb/Eu_ sample than for the singly doped XG_Tb_ one. Simultaneously, an increase in luminescence intensity within the yellowish-orange as well as red ranges was observed, and—compared with emissions recorded for the XG_Tb_ xerogel—the maxima of these bands were slightly shifted (from 580 nm to 584 nm, and from 618 nm to 616 nm). Thus, based on this observation, we could conclude that the indicated shift is a result of the superimposition of the yellow (^5^D_4_ → ^7^F_4_, 580 nm) and red band (^5^D_4_ → ^7^F_3_, 618 nm) of Tb^3+^ ions with orange (^5^D_0_ → ^7^F_1_, 590 nm) and red (^5^D_0_ → ^7^F_2_, 612 nm) luminescence originating from Eu^3+^.

**Figure 5:**
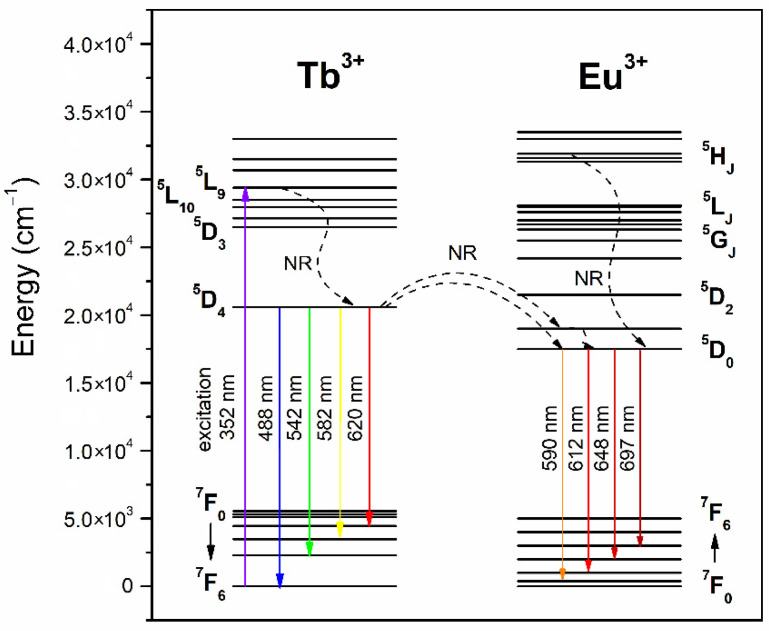
Energy level scheme of Tb^3+^ and Eu^3+^ ions.

Hence, our experimental results indicate the occurrence of Tb^3+^/Eu^3+^ ET upon excitation at a 352 nm wavelength when Tb^3+^ ions are excited from the ^7^F_6_ ground state. Then, the electrons at the ^5^L_9_ level decay rapidly through the ^5^G_5_, ^5^L_10_, and ^5^D_3_ states by the multiphonon relaxation process until the ^5^D_4_ level is populated. Since there is the energetical resemblance of the ^5^D_4_ (Tb^3+^) and the ^5^D_1_/^5^D_0_ (Eu^3+^) levels, the Tb^3+^/Eu^3+^ energy migration is feasible, and the excitation energy is transferred from Tb^3+^ to the adjacent Eu^3+^ ion. The acceptor ions relax from the ^5^D_0_ state to the ^7^F_J_ levels, promoting the light emission within the reddish-orange spectral region [43]. The ET is schematized in the level diagram presented in Figure 5.

The decay curves were registered for the green light at 541 nm, upon excitation at 352 nm from the near-UV range (inset in Figure 4b). For xerogels, a mono-exponential fit was used to evaluate the lifetimes of Tb^3+^, and the fitted curves are marked with a black line, while the collected experimental data are shown as green and blue lines for XG_Tb_ and XG_Tb/Eu_, respectively. A slight shortening in the decay time of the ^5^D_4_ (Tb^3+^) state from 1.11 ms (XG_Tb_) to 0.88 ms (XG_Tb/Eu_) was identified. An indicated decline in a lifetime for co-doped xerogel could be explained by introducing an additional decay pathway via Eu^3+^ ions. Indeed, the ET from Tb^3+^ to Eu^3+^ enhances the decay rate of the excited Tb^3+^ ions, resulting in the shortening of the ^5^D_4_ (Tb^3+^) lifetime. Hence, the analysis of luminescence decay curves also enables calculation of the efficiency of Tb^3+^/Eu^3+^ ET, based on the following equation [44]:

(3) $$\eta_{\mathrm{ET}}=\left(1-\frac{\tau }{\tau_{0}}\right)\cdot 100\%.$$

where τ_0_ and τ are the lifetimes of the ^5^D_4_ (Tb^3+^) state for sample singly doped with Tb^3+^, and the sample co-doped with Tb^3+^, Eu^3+^ ions, respectively. In the case of the studied xerogels, the efficiency of Tb^3+^/Eu^3+^ ET was estimated to be about 21%, and the comparable values were denoted for, e.g., fluoroborate glass (η_ET_ = 20%) [45].

### 3.4. Luminescence of SiO_2_-BaF_2_ Nano-Glass-Ceramics

**Figure 6:**
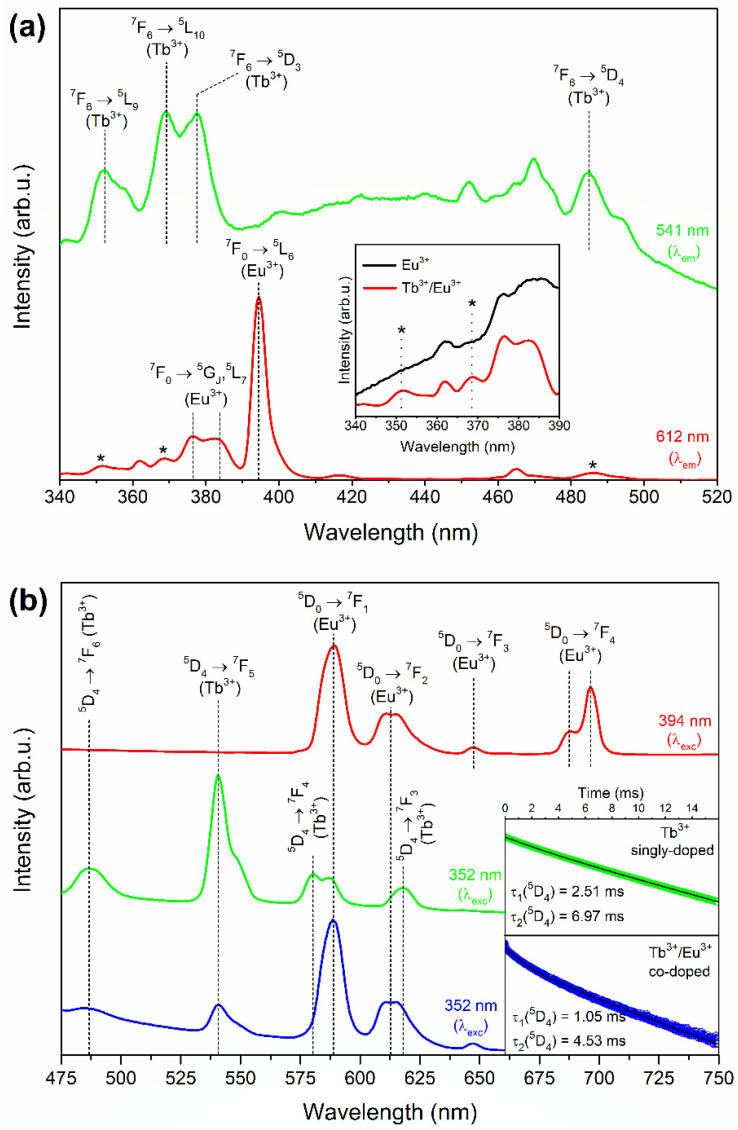
The excitation spectra recorded for Tb^3+^ (λ_em_ = 541 nm) and Eu^3+^ (λ_em_ = 612 nm) ions in prepared SiO_2_-BaF_2_ nano-glass-ceramics. For the latter, the additional lines originated from Tb^3+^ ions were marked by asterisks (**a**). The registered emission spectra for nGC_Tb_ (green line, λ_exc_ = 352 nm) as well as nGC_Tb/Eu_ glass-ceramics (blue line, λ_exc_ = 352 nm; red line, λ_exc_ = 394 nm). Inset shows the decay curves recorded for the ^5^D_4_ (Tb^3+^) state in nano-glass-ceramics (**b**).

The excitation spectra recorded for the nGC_Tb/Eu_ sample are shown in Figure 6a. The spectra emerged by monitoring the green luminescence characteristic for Tb^3+^ (541 nm), and the red emission originating from Eu^3+^ ions (612 nm). The luminescence of Tb^3+^ ions (541 nm) could be efficiently excited by the following wavelengths from the near-UV scope: 352 nm (^7^F_6_ → ^5^L_9_), 369 nm (^7^F_6_ → ^5^L_10_), and 377 nm (^7^F_6_ → ^5^D_3_), as well as from the VIS range: 485 nm (^7^F_6_ → ^5^D_4_). In the case of the excitation spectrum recorded at a 612 nm emission wavelength, an intense line appeared at 394 nm (^7^F_0_ → ^5^L_6_, Eu^3+^), but a few weaker bands at 376 nm (^7^F_0_ → ^5^G_J_), 384 nm (^7^F_0_ → ^5^L_7_), 418 nm (^7^F_0_ → ^5^D_3_), and 465 nm (^7^F_0_ → ^5^D_2_) were also detected. Similarly, as for xerogels, the recorded additional excitation lines at 352 nm, 369 nm, and 485 nm—marked in Figure 6a by asterisks—are typical for the ^7^F_6_ → ^5^L_9,10_, ^5^D_4_ transitions of Tb^3+^ ions, which could suggest the occurrence of Tb^3+^/Eu^3+^ ET in the studied nano-glass-ceramic samples. Similar results were found for other Tb^3+^, Eu^3+^ co-doped fluoride-based optical systems, e.g., pure CaF_2_ nanocrystals [46], and glass-ceramics containing SrF_2_ [47], as well as NaYF_4_ nanocrystals [48]. 

Figure 6b depicts the emission spectra collected for nGC_Tb/Eu_ and nGC_Tb_ samples, recorded upon excitation at 352 nm (a blue line for nGC_Tb/Eu_, and a green line for nGC_Tb_) and 394 nm (a red line) wavelengths. An excitation of the nGC_Tb_ sample using 352 nm results in registration of the visible emissions ascribed to the ^5^D_4_ → ^7^F_6_ (487 nm), ^5^D_4_ → ^7^F_5_ (541 nm), ^5^D_4_ → ^7^F_4_ (580 nm, 587 nm), and ^5^D_4_ → ^7^F_3_ (619 nm) transitions characteristic for Tb^3+^ ions. Subsequently, when the nGC_Tb/Eu_ co-doped sample was excited by a 394 nm wavelength, the luminescence bands originating from Eu^3+^ ions centered at 589 nm (^5^D_0_ → ^7^F_1_), 611 nm/614 nm (^5^D_0_ → ^7^F_2_), 648 nm (^5^D_0_ → ^7^F_3_), and 688 nm/696 nm (^5^D_0_ → ^7^F_4_) were observed. One can see that, in contrast to xerogel, the ^5^D_0_ → ^7^F_1_ magnetic dipole transition dominates the spectrum, which indicates that Eu^3+^ ions are placed at sites close to an inversion symmetry [49]. According to the calculated R/O ratio value (3.92 for XG_Tb/Eu_ and 0.51 for nGC_Tb/Eu_) and the literature [18], the observed change in emission profile clearly suggests that Eu^3+^ ions tend to embed into the BaF_2_ fluoride nanocrystal lattice by substituting Ba^2+^ cations. The decrease in the R/O ratio value was denoted for other Eu^3+^-doped oxyfluoride glass-ceramic systems described in the literature [50,51,52]. 

The emission spectrum of the nGC_Tb/Eu_ sample, collected upon 352 nm excitation, revealed an intense orange (589 nm) and red (611 nm/615 nm, and 647 nm) luminescence corresponding to the transitions of Eu^3+^ from the ^5^D_0_ level. Along with those bands, two emission lines with relatively low intensity were found within the blue–green scope and were assigned to the emissions originating from the ^5^D_4_ state of Tb^3+^ ions. Therefore, compared with XG_Tb/Eu_, the luminescence in the reddish-orange spectral range is particularly enhanced for nGC_Tb/Eu_. Based on this observation, we could conclude that the distance between interacting Tb^3+^ and Eu^3+^ ions in the prepared nano-glass-ceramics might be significantly shorter than in xerogels. Such shortening in the inter-ionic distance, strictly related to the segregation of rare earths inside BaF_2_ nanocrystals precipitated at 350 °C, could be responsible for a more efficient transfer of excitation energy from Tb^3+^ to Eu^3+^ ions. 

For the SiO_2_-BaF_2_ nano-glass-ceramics, the luminescence decay from the ^5^D_4_ level follows a double-exponential function with two different decay lifetimes. It results from the distribution of RE^3+^ ions between either the sol–gel host (described by faster τ_1_ component) and BaF_2_ nanocrystals (described by longer τ_2_ lifetime). The results are presented in the inset of Figure 6b, and the fitted decay curves are labeled with a black line, whereas the experimental data are tagged as green and blue lines for nGC_Tb_ and nGC_Tb/Eu_, respectively. For the sample singly doped with Tb^3+^ ions, the lifetime components are equal to τ_1_ = 2.51 ms and τ_2_ = 6.97 ms, while for the sample co-doped with Tb^3+^, Eu^3+^ the decay times are equal to τ_1_ = 1.05 ms and τ_2_ = 4.53 ms. Based on lifetime components, the average decay times, τ_avg_, were calculated from the following formula [53]:

(4) $$\tau_{\mathrm{avg}}=\frac{A_{1}\tau_{1}^{2}+A_{2}\tau_{2}^{2}}{A_{1}\tau_{1}+A_{2}\tau_{2}}.$$

Thus, the average luminescence lifetime of the ^5^D_4_ (Tb^3+^) state for nGC_Tb/Eu_ was determined to be τ_avg_ = 4.06 ms, and for nGC_Tb_ it equaled τ_avg_ = 6.56 ms. The analysis of luminescence decay curves showed a noticeable prolongation in lifetimes for SiO_2_-BaF_2_ nano-glass-ceramics compared with xerogels. It suggests that the amount of OH groups characterized by high vibrational energy (>3000 cm^−1^) should be significantly reduced in glass-ceramics. Moreover, the dopant ions tend to enter into the BaF_2_ nanocrystals characterized by low phonon energy (~319 cm^−1^ [54]), making the radiative relaxation from the ^5^D_4_ level more prominent compared with xerogels.

Additionally, based on luminescence lifetimes, the calculated ET efficiency for prepared SiO_2_-BaF_2_ nano-glass-ceramic exceeds 38%. In such a case, the distance between interacting RE^3+^ ions entering into BaF_2_ nanocrystals decreased, resulting in a reinforced transfer of energy absorbed by Tb^3+^ to Eu^3+^. Indeed, it is related to creating an energy transfer net among the donor and acceptor ions, causing the ET to become more frequent. Comparable values of ET efficiency were described for glass-ceramics containing YF_3_:1Tb^3+^, 0.5Eu^3+^ (mol.%) nanophase (η_ET_ ≈ 39%) [55].

Summarizing, due to the unique properties of BaF_2_, e.g., a broad region of transparency from 0.14 μm up to 14 μm, wide bandgap (11 eV), and low maximum phonon energy (~319 cm^−1^), the oxyfluoride glass-ceramics containing BaF_2_ nanophase are extensively applied to generate an efficient up- [11] and down-conversion luminescence [9], or white light emission [20]. Therefore, such materials could be successfully used for laser technologies, spectral converters, and three-dimensional displays [10]. Since Eu^3+^ ions emit within the red or reddish-orange light area, and Tb^3+^ ions are well known as green emitters, the fabricated SiO_2_-BaF_2_:Tb^3+^, Eu^3+^ nano-glass-ceramics are able to generate multicolor luminescence. Thus, sol–gel materials might be considered for use as optical elements in RGB lighting optoelectronic devices operating upon near-UV excitation. 

## 4. Conclusions

This work presented the fabrication of Tb^3+^, Eu^3+^ co-doped oxyfluoride glass-ceramics at 350 °C from xerogels prepared via the sol–gel technique. The analysis of the thermal behavior of xerogels was performed using TG/DSC measurements, and the structural properties were determined based on ATR-IR spectroscopy. The crystallization of BaF_2_ at the nanoscale was confirmed by XRD and TEM measurements. The characterization of sol–gel samples involved an excitation of the prepared sol–gel materials upon near-UV irradiation at 352 nm which showed the Tb^3+^/Eu^3+^ energy transfer, resulting in strengthening the luminescence within the reddish-orange light scope due to additional emission from Eu^3+^ ions. Nevertheless, for xerogels, the blue–green luminescence (^5^D_4_ → ^7^F_5,6_ of Tb^3+^) dominated, meanwhile, the reddish-orange emission (^5^D_0_ → ^7^F_0–4_ of Eu^3+^ overlapped with ^5^D_4_ → ^7^F_4,3_ bands of Tb^3+^) was particularly enhanced for SiO_2_-BaF_2_ nano-glass-ceramics. The luminescence decay kinetics showed that in the co-doped sol–gel materials, the energy transfer from Tb^3+^ to Eu^3+^ ions occurred with an efficiency that varied from 21% for xerogels to 38% for nano-glass-ceramics. An indicated increase in energy transfer efficiency for prepared nano-glass-ceramics could be explained by shortening the distance between interacting Tb^3+^ and Eu^3+^ ions embedded into the BaF_2_ nanocrystal lattice. The obtained results suggest that the fabricated SiO_2_-BaF_2_:Tb^3+^, Eu^3+^ nano-glass-ceramics could be predisposed to application in selected technologies, e.g., three-dimensional displays and color screens.

## Figures and Tables

**Table 1:** The parameters from TG analysis for studied sol–gel materials.

Sample	Number of Degradation Steps	Temperature Range (°C)	Weight Loss (%)
XG_Tb_	1st	45–208	2.75
	2nd	208–322	17.56
XG_Tb/Eu_	1st	45–204	3.56
	2nd	204–321	17.54

**Table 2:** The parameters from DCS curves recorded for fabricated silicate sol–gel samples.

Sample	Peak Maximum (°C)	Exchanged Heat (J/g)
XG_Tb_	305	−118.3
XG_Tb/Eu_	306	−117.9

## Data Availability

The data presented in this study are available on request from the corresponding authors.
